# Effectiveness of Expressive Writing in the Reduction of Psychological Distress During the COVID-19 Pandemic: A Randomized Controlled Trial

**DOI:** 10.3389/fpsyg.2020.587282

**Published:** 2020-11-10

**Authors:** Maša Vukčević Marković, Jovana Bjekić, Stefan Priebe

**Affiliations:** ^1^Department of Psychology, Faculty of Philosophy, University of Belgrade, Belgrade, Serbia; ^2^Psychosocial Innovation Network (PIN), Belgrade, Serbia; ^3^Institute for Medical Research, University of Belgrade, Belgrade, Serbia; ^4^Unit for Social and Community Psychiatry (World Health Organization Collaborating Centre for Mental Health Services Development), Queen Mary University of London, London, United Kingdom

**Keywords:** expressive writing, online intervention, psychological distress, depression, anxiety, stress, well-being, mental health intervention (MeSH)

## Abstract

**Objective:**

Due to the wide impact of the COVID-19 pandemic on mental health, the need for scalable interventions that can effectively reduce psychological distress has been recognized. Expressive writing (EW) can be beneficial for different conditions, including depression, suicidal ideation, and coping with trauma. Therefore, we aim to assess the applicability and effectiveness of an online format of EW in the reduction of psychological distress in context of the COVID-19 pandemic.

**Methods:**

In this parallel-group, randomized controlled trial, participants (*n* = 120) were randomly allocated to (1) the intervention group-who completed five EW sessions over the 2 week period-or (2) the control group-who received treatment as usual (TAU). Participants were assessed for primary and secondary outcome measures at baseline, post-treatment, and follow-up-1-month after the treatment. The primary outcome was severity of psychological distress assessed at post-treatment, operationalized as Depression Anxiety Stress Scale (DASS) summary score. Secondary outcomes were severity of depression, anxiety, and stress (DASS subscale scores), well-being (WHO-5), subjective perception of quality of life (SQOL), and subjective evaluation of difficulties coping with pandemic, which were also assessed at post-treatment. Per protocol, analysis was conducted with available cases only.

**Results:**

A less favorable outcome was found in the intervention group on psychological distress, and symptoms of stress, after controlling for baseline scores. Increased stress was recorded in the treatment group, with no effect in the control group. There was no significant difference between the groups on depression, anxiety, well-being, and subjective quality of life. No group effect for any of the outcomes measures was recorded at follow-up. Additional analysis revealed moderation effects of age and gender with older and male participants scoring higher on distress measures.

**Conclusion:**

Engaging in EW during the pandemic was found to elevate stress; thus, when applied in the context of the COVID-19 pandemic, it may be harmful. Hence, EW or similar self-guided interventions should not be applied without prior evidence on their effects in the context of a pandemic and similar stressful and unpredictable circumstances.

**Clinical Trial Registration:**

This study is approved by the Institutional Ethics Committee (Protocol number #2020-20), and a trial has been registered at ISRCTN registry https://www.isrctn.com/ISRCTN17898730.

## Introduction

The current COVID-19 pandemic brought numerous physical and mental health risks, which have been shown to lead to moderate to severe depression, anxiety, and traumatic stress-related difficulties in the general population (Wang et al., 2020). One study found that the prevalence of depression symptoms among US adults is threefold higher during the COVID-19 pandemic than before (Ettman et al., 2020). Similar trends were reported by a researcher from Hong Kong who found that a quarter of participants reported deteriorated mental health due to the pandemic, with elevated levels of depression and anxiety (Choi et al., 2020). In addition to the pandemic itself, various measures for the prevention and spread of COVID-19 have both short- and long-term negative impacts on mental health and well-being (Brooks et al., 2020). Finally, the negative social and economic impacts of the pandemic are expected, which represent additional risk factors for mental health. These multifactorial and complex effects should be expected to persist for a long period of time after the pandemic is over. To prevent and mitigate the negative effects of the COVID-19 pandemic, it was recognized that the development and implementation of mental health programs, including assessment, support, and treatment should be prioritized (Xiang et al., 2020).

With limited resources and the additional burden put on the public health care system during the pandemic, as well as restricted possibilities for the usage of traditional mental health services due to measures for prevention of COVID-19, such as physical distancing, there is a need for novel approaches, strategies, and interventions that reduce the short- and long-term negative psychological effects of the pandemic. Furthermore, the wide spread and the duration of the COVID-19 pandemic brought additional challenges to the mental health system as the number of people in need of psychosocial support increased beyond the capacities of mental health units even in the most developed countries. That is, there is a need for mental health interventions that are applicable to a large number of people, i.e., members of the general population without a history of mental health difficulties, who are experiencing pandemic-related psychological distress.

### Expressive Writing

Expressive writing (EW) is an intervention in which one is asked to disclose one’s deepest thoughts and feelings surrounding a stressful life event, initially introduced by Pennebaker in 1986 (Pennebaker and Chung, 2012; Andersson and Conley, 2013). The idea behind EW is that one could decrease negative feelings, and improve physical and mental health, by engaging in deep and meaningful writing about a traumatic or difficult event. EW is supposed to provide a person with a safe environment in which to reflect, explore their feelings, and integrate the difficult and hurtful experience. This idea received support in early studies, which showed that EW can be beneficial for the improvement of psychological and physical health (Smyth, 1998). Furthermore, EW was found to reduce medical visits (Pennebaker and Francis, 1996). Although there is no single and unique underlying mechanism or explanation on how EW leads to improved health, numerous complementary theories, including disinhibition of emotions, cognitive adaptation and reorganization, enhanced emotion regulation, exposure to aversive stimuli, and re-experiencing events and habituation to emotional stimuli, have been proposed to date (Pennebaker and Chung, 2012; Perry and Ward-Smith, 2018; Sabo Mordechay et al., 2019).

Within the past 30 years, there have been numerous studies that assessed if EW is beneficial for physical and mental health. These studies included a broad variety of samples, settings, EW instructions and outcomes, and overall yielded mixed findings. Despite the overall inconsistent results, studies have shown that EW has benefits for mood-related psychological difficulties. Namely, studies have shown that EW reduces depressive symptoms in both general and at-risk populations (Gortner et al., 2006; Sloan et al., 2008). Furthermore, EW has proven beneficial for people reporting high levels of depression and anxiety (Graf et al., 2008).

To systematize this broad and complex literature, several meta-analyses examining the effects of EW have been conducted over the years. Unfortunately, the results were inconclusive even at the meta-analytic level. Namely, an early meta-analysis found an overall positive effect of EW and concluded that it has comparable effects to other psychological treatments (Smyth, 1998). Later, Fristina and colleagues (Frisina et al., 2004) found a small but significant effect for physical health outcomes, but no effect on psychological outcomes. More recently, several meta-analytic studies have not found supporting evidence for the effectiveness of EW for either physical or mental health (Meads and Nouwen, 2005; Mogk et al., 2006; Reinhold et al., 2018), except for the reduction of posttraumatic stress difficulties (Pavlacic et al., 2019). On the contrary, the largest and the most inclusive meta-analysis to date (Frattaroli, 2006) found a significant positive overall effect of EW. More specifically, he found significant average effect for reducing symptoms of depression (unweighted effect size *r* = 0.073), as well as for distress (unweighted effect size *r* = 0.102) and anxiety (unweighted effect size *r* = 0.051). It is important to note that all these meta-analyses had different study-inclusion criteria and differed in regard to the analytic approach they employed. Looking beyond the inconsistent findings, the meta-analytic studies highlighted the disparity in the quality between the trials and brought to light several important moderating variables that can affect the outcome of the EW intervention.

A close examination of factors contributing to EW efficiency revealed that the more specific the EW intervention is, the greater the chance it will have beneficial results (Reinhold et al., 2018). Moreover, EW was found to be more effective when the number of writing sessions was higher (Frattaroli, 2006; Reinhold et al., 2018), when sessions were longer, and when instructions were more directive, or included a specific writing topic (Reinhold et al., 2018). Some authors discussed the importance of the moderating effect of specificity of writing instructions, since it has been shown that more specific writing instructions are especially valuable for people with certain mental health conditions (e.g., depression), due to which they are experiencing distress as it enables them to adhere to EW requirements more effectively (Baum and Rude, 2013; Rude and Haner, 2018).

Studies exploring individual differences in responsiveness and factors that contribute to the positive effects of EW indicated that participants who perceive their stressful event as more severe benefit more from EW (Greenberg and Stone, 1992). In addition, those who experience moderate severity of negative emotions and are more aware of negative feelings gain the most from EW (Norman et al., 2004; Sabo Mordechay et al., 2019). It seems as if experiencing too many or too few negative feelings can interfere with the underlying processes required for an EW intervention to be beneficial (Sabo Mordechay et al., 2019). Finally, EW intervention group-level effects are stronger when there is a higher percentage of females and a higher mean age of participants in the sample (Reinhold et al., 2018).

### Promises of EW in the Context of COVID-19 Pandemic

There is a significant body of evidence to support potential positive effects of EW interventions, and there are various practical benefits of the application of EW in the context of the current COVID-19 pandemic: it can be easily administered, is self-guided, does not require any additional resources, does not present an additional burden for the health system, and can be delivered remotely. EW can be easily adapted for online delivery and has shown positive effects even in an online modality (see Karen et al., 2012). With limited resources and constraints of the health care system during the pandemic, as well as restricted possibilities for the usage of traditional mental health services due to measures for the prevention of COVID-19, the need for cost-effective mental health interventions, such as EW, which could be applied to the large number of people experiencing pandemic-related psychological distress, became even more important.

### Current Study

This study aims to assess the effectiveness of EW interventions in the reduction of psychological distress in the general population during the COVID-19 pandemic by conducting a randomized controlled trial (RCT). We assess if applying a fully remote (online) EW intervention is more effective at reducing psychological distress in the general population than receiving treatment as usual (TAU), which is commonly advised under these circumstances. In addition to measuring effectiveness in the reduction of psychological distress, effects on improving positive aspects of psychological functioning including well-being and satisfaction with quality of life will be assessed. We hypothesized that receiving the EW intervention would be more effective in the reduction of psychological distress and improvement of well-being and satisfaction with quality of life than receiving TAU. If proven to lead to the reduction of psychological distress, EW interventions could be further applied and explored in different settings, including a potential *next wave* of the COVID-19 pandemic, other emergency settings, and in countries in which the health care systems have limited access to mental health care due to specific circumstances (e.g., civic unrest or war) or lack of resources.

## Materials and Methods

### Study Design and Participants

In this parallel-group RCT, participants were recruited through a social media advert. The advert included information regarding the opportunity to be involved in a study assessing the effectiveness of an online intervention aiming to reduce the psychological distress people may experience during the COVID-19 pandemic, and an invitation for potential participants to join the study by signing up online. Those who signed up were provided with a written explanation about the study, activities to be performed, and what would be expected of them if they decide to sign up. Furthermore, potential participants were informed about the inclusion criteria for the study and asked to complete a self-assessed eligibility questionnaire for the following criteria: (a) minimum 18 years of age, (b) native Serbian speaker, and (c) willing to provide informed consent. Eligible participants who decided to participate were then asked to sign an informed consent form and leave an email address to be used for all further correspondence. Those who met the criteria and decided to sign the consent form were contacted by Researcher 1 (MVM) who provided an additional explanation about the study, collected demographic information and COVID-19-related experiences data, and conducted baseline assessments before randomization. This study was approved by the Institutional Ethics Committee (Protocol number #2020-20), and the trial has been registered at ISRCTN registry https://www.isrctn.com/ISRCTN17898730.

### Randomization and Masking

Participants were randomly allocated (1:1) either to the intervention group-who received the EW intervention-or the control group-who received TAU, i.e., informal support through families, friends, and networks (face-to-face, telephone, and online) as well as support from available services in the community during the state of emergency (e.g., online counseling, hotlines, available self-help manuals). Randomization was performed by Researcher 2 (JB) who used the web-based system Research Randomizer (Version 4.0) (Urbaniak and Plous, 2013) for random allocation of the participants into two groups. Following the randomization, each participant received a personalized information sheet containing a schedule of the upcoming activities they would be participating in. The group allocation was not disclosed to participants.

Researcher 1, who assessed outcome measures, was also blind to the allocation of participants. Researcher 2, who performed the randomization, was blind to the baseline assessment results. Researcher 3 (SP), who managed the overall supervision of study implementation, was blind to both the outcome measure results and the allocation of participants.

### Procedure

The whole trial was conducted online, without any in-person contact between participants and researchers, or among participants. The questionnaires were emailed to participants using an online custom survey platform, and the EW intervention was implemented using the same online platform. Participants allocated to the intervention group were assigned to complete five EW sessions, each lasting 20 min. The EW sessions were set 3 days apart, over a 2 weeks period. Prior to the first session, participants were emailed a brief explanation about EW and their expected engagement. More specifically, the participants were informed about the expected number of EW sessions and the conditions under which they are expected to write (i.e., that they need to be alone in the room, in a place they feel comfortable, to ensure that they have set aside enough time to complete the activity without any distractions, and to shut down all devices and notifications during witting). During each session, participants received the following instruction: *During the next 20 min, write about any experiences and thoughts on your life during the pandemic, write everything that comes to your mind and try to follow your thoughts as they come to you. Feel free to write everything that comes to your mind; don’t read back, delete, or change your text, simply write your thoughts, and don’t stop for 20 min*. This instruction was visible to participants at all times as they were writing. Participants allocated to the control group received TAU, i.e., informal support through families, friends, and networks. As this trial was conducted under the highly unpredictable and uncontrollable circumstances of the COVID-19 pandemic, we opted for a “natural” control group, i.e., people who were using different available resources to improve their mental health other than EW.

Outcome measures were assessed at three time points-at baseline, post-treatment (a day after the last EW intervention), and follow-up (1-month after the intervention has ended). Information on the number of EW interventions completed by each participant was also registered. Demographic information was collected during the baseline assessment, immediately before randomization, while information about additional support and experiences with the EW intervention was collected during the follow-up assessment.

At the end of the study, all participants were offered to receive feedback based on their baseline and post-assessment results and information on available services offering free psychological support and self-help materials provided by either the government or specialized institutions/organizations aimed at protection of psychological well-being during the pandemic.

All participants’ identifiable data were password-protected and accessible only to Researcher 2. All data were entered into an SPSS database and were anonymized before being shared with other researchers and retrieved for data quality inspection.

### Outcomes

The primary outcome was the severity of psychological distress, assessed using DASS 21-Depression Anxiety Stress Scale, short version (Osman et al., 2012), measured at post-treatment. Secondary outcomes assessed at post-treatment were severity of depression-related psychological distress, assessed using DASS 21 depression subscale; anxiety-related psychological distress, assessed using DASS 21 anxiety subscale; stress-related psychological distress, assessed using DASS 21 stress subscale; well-being, assessed using the WHO well-being index; and subjective perception of the quality of life, assessed using the SQOL, the mean score of the 12 satisfaction items from the Manchester Short Assessment of Quality of Life (MANSA) (Priebe et al., 1999). DASS 21 was selected as a primary outcome for several reasons. First, it enables the capture of the most prominent difficulties that can be expected in a pandemic-depression, anxiety, and stress. Furthermore, this instrument has good psychometric properties with internal consistency >0.85 (Cronbach’s alpha), which tends to be stable across different countries i.e., Greece (Lyrakos et al., 2011), Turkey (Zanan and Nuran, 2010), Nigeria (Coker et al., 2018), Vietnam (Le et al., 2017), Brazil (Batistelli Vignolaa and Marcassa Tucci, 2014), etc. The Serbian version of DASS21 showed high validity and reliability with internal consistency coefficients, i.e., Cronbach’s alpha = 0.87, 0.82, and 0.86 for depression, anxiety, and stress, respectively (Batistelli Vignolaa and Marcassa Tucci, 2014). Moreover, this instrument was selected as it can be administered online for both clinical and research purposes (Lovibond and Lovibond, 1995). Finally, due to the fact that DASS 21 does not measure traits, but psychological states, it can be expected to validly capture changes in one’s emotional state over a relatively short period of time (Lovibond and Lovibond, 1995). In addition to DASS 21, we used MANSA to capture positive aspects of psychological functioning. MANSA has good psychometric properties, considering it is a brief measure (Cronbach’s alpha = 0.74), and a high correlation with subjective quality of life assessments (Priebe et al., 1999).

In addition to the outcome measures, basic demographic information, satisfaction with social support including satisfaction with personal relationships and satisfaction with support of friends (Atroszko et al., 2015), and information on COVID-19-related experiences (e.g., if they or members of their family are diagnosed with COVID-19, if they were experiencing symptoms, etc.) were collected at baseline. Data regarding potential usage of any other psychosocial support services (e.g., online counseling, reading self-help manuals and guidance on how to cope during the state of emergency, social media blogs on emotion regulation, etc.) were collected at both post-treatment and the follow-up assessment. Finally, at the follow-up assessment, participants from the intervention group were asked about their experiences related to the EW intervention: (1) if they felt as if it was useful and (2) if it was too much of a burden.

### Statistical Analyses

The sample size was determined using G^∗^Power 3.1 software with the expected effect sizes (η^2^ of 0.06 and the power of 0.80 in respect to planned statistical analysis-ANCOVA). This expectation about the effect size was based on previous EW studies (Mogk et al., 2006; Pavlacic et al., 2019; Reinhold et al., 2018). The statistical analysis plan was defined before unblinding the data or conducting any analysis, and all statistical analyses were carried out in line with the statistical analysis plan. All analyses were done using IBM SPSS Statistics for Windows, Version 22.0. Post-treatment outcomes were compared using general linear models, adjusting for the baseline score of the given outcome, i.e., analysis of covariance (ANCOVA). For post-treatment outcome, variables at post-treatment were entered as dependent, Group (EW vs. TAU) was a between-subject factor, and baseline score was entered as a covariate to control for individual differences at baseline. Follow-up data were analyzed in the same manner with outcome score at 1-month follow-up as the independent variable. Finally, to assess the effects of personal characteristics that could moderate the effects, we performed interaction analysis (Group × Gender; Group × Age Group) with baseline-to-post-treatment change score as a dependent variable. All analyses were done with available cases only. Given the exploratory nature of the study, per protocol analysis included only participants who completed treatment, defined as completing a minimum of four out of five EW sessions, while excluding participants based on the following criteria: if they experienced the death of a family member or close friend during the trial, and if they were experiencing severe mental health distress, defined as scoring 3 SD above average on the DASS at either the pre- or post-test. Significance testing was set at the 5% level, across all analyses.

## Results

**FIGURE 1:**
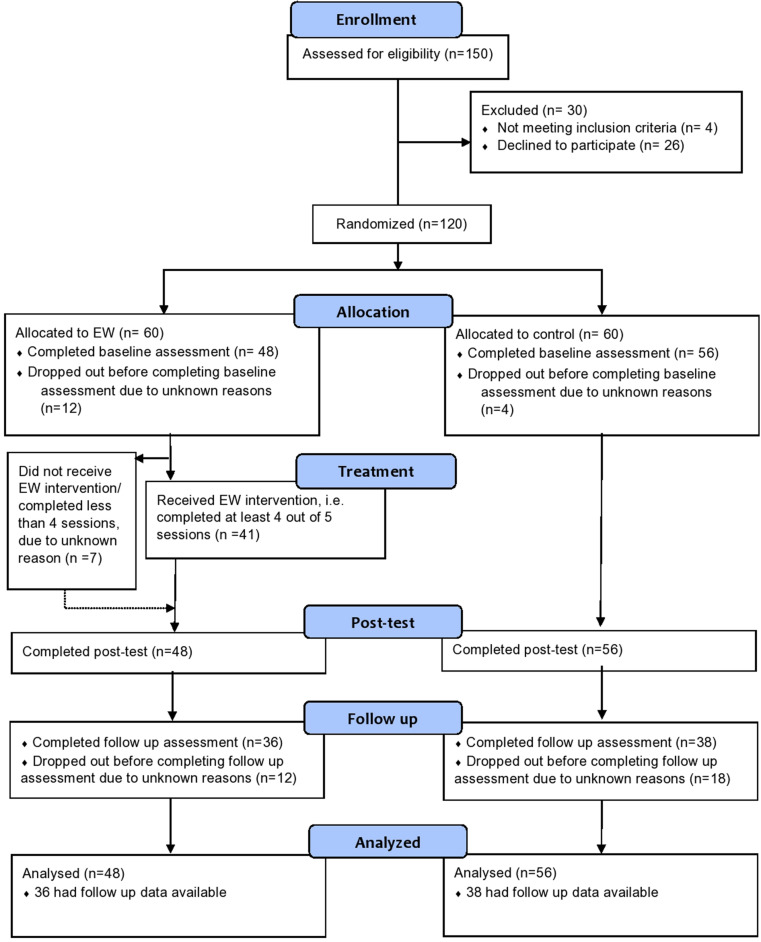
Trial flow chart.

During the recruitment period (7 to 14 of April), 150 participants signed up for the study, out of which 120 were randomized across EW and TAU groups. Complete data at post-treatment were obtained for 104 participants while 74 participants were assessed at follow-up (Figure 1).

Demographic characteristics were balanced between the EW and control groups (Table 1). There were more female than male participants in both groups. All participants were symptom-free and not tested for COVID-19, while very few had had close contact (i.e., family members or close friends) with confirmed COVID-19 contagion (five in the treatment group and three in the control group). All demographic and COVID-related information about the sample is presented in Table 1.

**TABLE 1:** Demographic characteristics of the sample.

**Variable**	**Treatment**	**Control**
**Gender**		
Male [%]	23%	30%
Female [%]	77%	70%
Age [years; mean (SD)]	31.79 (9.062)	32.67 (10.848)
Education [years; mean (SD)]	15.69 (3.088)	15.75 (2.480)
**Do you have a chronic illness?**		
Yes [%]	21	7
No [%]	67	84
Not sure [%]	2	4
**Are you doing a job that requires you to be physically in contact/close to more than a few people?**		
Yes [%]	49	30
No [%]	51	70
**Are you working in the health sector (e.g., as a nurse, medical doctor, etc.)?**		
Yes [%]	2	2
No [%]	98	98
**Are you at risk of losing your job or experiencing a significant decrease in income?**		
Yes [%]	35	36
No [%]	65	64
**Are you or have you been infected with the novel coronavirus?**		
Yes [%]	0	0
No [%]	100	100
**Have members of your family or your close friends been infected with the novel coronavirus?**		
Yes [%]	16	11
No [%]	88	89
How satisfied are you with your personal relationships? [9-point scale; mean (SD)]	6.95 (1.786)	7.36 (1.545)
How satisfied are you with the support you get from your friends? [9-point scale; mean (SD)]	7.58 (1.367)	7.71 (1.513)
How would you rate your knowledge level on the novel coronavirus? [9-point scale; mean (SD)]	6.47 (1.609)	6.55 (1.449)
How would you rate your knowledge level on how to prevent the spread of the novel coronavirus? [9-point scale; mean (SD)]	7.70 (1.036)	7.94 (1.406)
What is your probability of getting infected with the novel coronavirus? [9-point scale; mean (SD)]	4.42 (1.562)	3.77 (1.579)
How susceptible do you consider yourself to a novel coronavirus infection? [9-point scale; mean (SD)]	4.79 (1.833)	4.28 (2.051)
I follow the recommendations from authorities in my country to prevent spread of novel coronavirus. [9-point scale; mean (SD)]	8.44 (0.796)	8.36 (1.128)
**Usage of psychological support or counseling services**		
Yes [%]	21	9
No [%]	54	59
No response [%]	25	32
**Usage of self-help psychosocial support services (e.g., reading self-help manuals and guidance on how to cope during the state of emergency, social media blogs on emotion regulation, etc.)**		
Yes [%]	17	2
No [%]	58	66
No response [%]	25	32

Primary outcome data were available for 104 participants-48 participants in the treatment group and 56 participants in the control group. All primary and secondary outcome measures at all time points are summarized in Table 2. The comparison between baseline and post-treatment in the treatment group shows no difference in the primary outcome. The only statistically significant effect was observed in post-treatment stress level (Table 3). In contrast, in the control group, no differences were observed for any of the outcome measures between baseline and post-treatment. The baseline-to-follow-up comparison showed significant differences in primary outcome in both the treatment and control groups. For the secondary outcome measures, baseline-to-follow-up differences were observed for depression in both the treatment and control groups, as well as stress in the treatment group. The changes and the correlations between baseline and post-treatment as well as between baseline and follow-up for both primary and secondary outcomes are presented in Table 3.

**TABLE 2:** Descriptive statistics for primary and secondary outcomes for treatment and control groups across baseline, post-treatment, and follow-up.

	**Treatment**						**Control**					
**Outcomes**	**Baseline**		**Post-treatment**		**Follow-up**		**Baseline**		**Post-treatment**		**Follow-up**	
	**(*N* = 48)**		**(*N* = 48)**		**(*N* = 36)**		**(*N* = 56)**		**(*N* = 56)**		**(*N* = 38)**	
	***M***	***SD***	***M***	***SD***	***M***	***SD***	***M***	***SD***	***M***	***SD***	***M***	***SD***
DASS (Tot)	22.04	15.139	22.46	12.881	15.61	12.838	15.84	11.747	14.21	11.972	13.24	9.733
Depr (DASS)	6.21	5.750	6.25	5.105	3.83	4.339	4.66	4.837	4.05	4.534	3.55	4.065
Anxiety (DASS)	5.69	5.654	4.65	4.970	3.56	4.925	2.93	3.879	2.93	4.276	2.29	2.779
Stress (DASS)	10.15	5.986	11.56	5.124	8.22	5.509	8.25	4.944	7.23	5.250	7.39	5.405
WHO	3.13	0.844	3.16	0.863	3.35	0.816	3.14	0.822	3.29	0.829	3.26	0.842
SQOL	4.90	1.032	4.89	1.070	4.98	1.022	4.99	1.149	5.03	1.126	4.94	1.295
Hard time making it through the pandemic	2.08	1.007	2.19	1.065	–	–	1.696	0.761	1.57	0.759	–	–

**TABLE 3:** The changes and the correlations between baseline and posttreatment and baseline and follow up for both primary and secondary outcomes.

	**Treatment**				**Control**			
**Outcomes**	**Baseline – Post treatment (*N* = 48)**		**Baseline – Follow – up (*N* = 36)**		**Baseline – Post – treatment (*N* = 56)**		**Baseline – Follow – up (*N* = 38)**	
	**Difference**	***r***	**Difference**	***r***	**Difference**	***r***	**Difference**	***r***
DASS total	*t*(47) = –0.235, *p* = 0.815	0.628	*t*(35) = 2.398, *p* = 0.022	0.528	*t*(55) = 1.244, *p* = 0.219	0.661	*t*(37) = 2.115, *p* = 0.041	0.488
Depression (DASS)	*t*(47) = –0.058, *p* = 0.954	0.580	*t*(35) = 2.242, *p* = 0.031	0.529	*t*(55) = 1.253, *p* = 0.216	0.702	*t*(37) = 2.651, *p* = 0.012	0.588
Anxiety (DASS)	*t*(47) = 1.349, *p* = 0.184	0.499	*t*(35) = 1.946, *p* = 0.060	0.443	*t*(55) = 0.000, *p* = 1.000	0.618	*t*(37) = 1.467, *p* = 0.151	0.391
Stress (DASS)	*t*(47) = –2.290, *p* = 0.027*	0.713	*t*(35) = 2.078, *p* = 0.045	0.617	*t*(55) = 1.766, *p* = 0.083	0.644	*t*(37) = 1.330, *p* = 0.192	0.536
Well-being (WHO)	*t*(47) = –0.338, *p* = 0.737	0.755	*t*(35) = –1.983, *p* = 0.055	0.615	*t*(55) = –1.874, *p* = 0.066	0.711	*t*(37) = –1.849, *p* = 0.072	0.653
SQOL	*t*(47) = –0.165, *p* = 0.870	0.806	*t*(35) = –0.294, *p* = 0.770	0.794	*t*(55) = –0.449, *p* = 0.655	0.757	*t*(37) = 0.962, *p* = 0.342	0.854
Hard time making it through the pandemic	*t*(47) = –0.658, *p* = 0.514	0.441			*t*(55) = –1.308, *p* = 0.196	0.557		

The ANCOVA for treatment versus control group on primary outcome, i.e., post-test DASS total scores, controlling for baseline DASS score was found to have a statistically significant main effect of group *F*(1,101) = 5.600, *p* = 0.020, _*p*_η^2^ = 0.053. There was a significant effect of treatment on post-test stress after controlling for stress at baseline *F*(1,101) = 16.360, *p* = 0.000, _*p*_η^2^ = 0.139, with the treatment group scoring higher on both measures. A main effect of group on post-test Depression and Anxiety, after controlling for baseline scores, was not found: *F*(1,101) = 3.078, *p* = 0.082, _*p*_η^2^ = 0.030 and *F*(1,101) = 0.115, *p* = 0.735, _*p*_η^2^ = 0.001, respectively. A main effect of group on Well-being and Subjective quality of life post-test scores, when controlling for baseline scores on these scales, was not found either: *F*(1,101) = 1.276, *p* = 0.261, _*p*_η^2^ = 0.012 and *F*(1,101) = 0.352, *p* = 0.554, _*p*_η^2^ = 0.003 respectively. The ANCOVA for treatment versus control group on post-test measure of having a hard time making it through the coronavirus pandemic and state of emergency, when controlling for baseline score on this measure, showed a significant group effect, with the treatment group scoring significantly higher than the control group *F*(1,101) = 6.813, *p* = 0.010, _*p*_η^2^ = 0.063.

The ANCOVA for EW versus TAU group on follow-up outcome measures revealed no statistically significant main group effect when controlling for baseline scores. Specifically, no effect was found for DASS total score, *F*(1,71) = 0.087, *p* = 0.769, _*p*_η^2^ = 0.001, Depression, *F*(1,71) = 0.025, *p* = 0.874, _*p*_η^2^ = 0.000, Anxiety, *F*(1,71) = 0.416, *p* = 0.521, _*p*_η^2^ = 0.006, and Stress subscales, *F*(1,71) = 0.001, *p* = 0.970, _*p*_η^2^ = 0.000, Well-being, *F*(1,71) = 0.174, *p* = 0.678, _*p*_η^2^ = 0.002, and Subjective quality of life, *F*(1,71) = 0.691, *p* = 0.408, _*p*_η^2^ = 0.010.

Per protocol analysis resulted in the exclusion of seven participants from the treatment group based on treatment compliance as well as four participants from the experimental and three participants from the control group based on other criteria. Following the exclusion of 14 participants, we reran the analysis on post-treatment outcomes. Most of the results stayed the same, except for the effect on depression, which reached the significance threshold. Results of the complete, per protocol analysis are shown in Supplementary Material.

To explore the factors contributing to the outcome of the intervention, we calculated the CHANGE score for the main outcome measure (e.g., DASSchange = DASSpost − DASSpre), for which positive values indicate elevated symptoms and negative values indicate reduced symptoms. To assess if the change in the main outcome (DASS total score change) was different between the experimental and control groups depending on characteristics of the participants, we performed a series of interaction analyses-ANOVAs with DASS total score change as dependent variable and GROUP (treatment vs control) with GENDER (male vs. female) or AGE GROUP (younger vs. older; median split at 30 years with younger being those aged 30 and less) as predictors.

**FIGURE 2:**
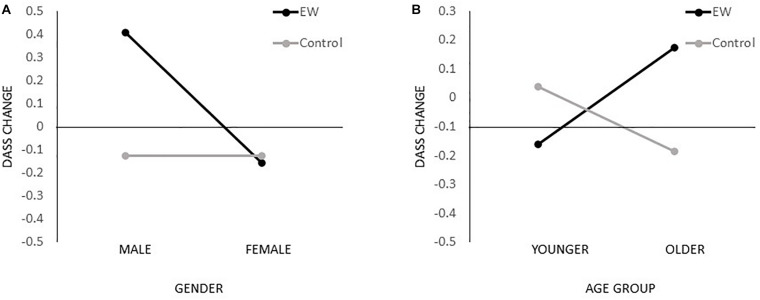
Interaction effects on the primary outcome. Interaction effects gender × treatment group **(A)** and age group × treatment group **(B)** for the primary outcome change score (DASSchange = DASSpost – DASSpre); positive values indicate elevated symptoms while negative values indicate reduced symptoms.

A significant GROUP × GENDER interaction effect was observed, *F*(1,92) = 6.989, *p* = 0.010, _*p*_η^2^ = 0.071, as well as a GROUP × AGE GROUP interaction, *F*(1,100) = 7.682, *p* = 0.007, _*p*_η^2^ = 0.071, indicating that EW interventions may be particularly counterproductive for older and male participants (Figure 2).

After the follow-up assessment, EW group participants were invited to share their perceptions and experiences of the intervention. Two-thirds of participants reported that participating in EW was beneficial for them personally. Only 6 out of 36 participants reported the EW intervention being time-consuming and difficult to complete.

## Discussion

This study assessed the effectiveness of a fully remote EW intervention in the reduction of psychological distress and improvement of well-being and satisfaction with the quality of life in the general population in the context of the COVID-19 pandemic. Our study found no evidence that five sessions of remote EW generate benefits in lowering depression, anxiety, and stress, and increasing overall well-being. On the contrary, our results showed that engaging in EW during the pandemic elevates the stress level of participants from the intervention group. The same results were obtained when controlling for the baseline results. Results of the follow-up assessment indicated that the severity of depressive and overall psychological distress measured by DASS total score decreased in both groups; however, no differences between groups were obtained. These results indicate that participation in the EW intervention did not have long-term effects. Per protocol analysis, which resulted in the exclusion of 14 participants, revealed similar results, with additional identified effects of EW on evaluated symptoms of depression in the treatment group. Finally, results indicated that EW intervention under these specific circumstances may be particularly counterproductive for older and male participants.

The rationale behind assessing the effectiveness of EW intervention was that, in the context of the pandemic, many people without a previous history of psychosocial difficulties may experience fear, anxiety, and depression. Moreover, the pandemic made access to mental health care more difficult as many primary health care institutions were either transformed into COVID-19 units or had to change the way they operate to comply with measures aiming to contain and limit the spread of the disease. Therefore, the current pandemic requires additional mental health care for the increased number of people in need and, at the same time, puts constraints on how psychosocial support can be provided. Hence, interventions like EW, which can be performed remotely and could address the needs of a large number of people with existing resources, seem a promising path to tackle this issue. Still, some limitations to our study need to be pointed out. First, the intervention aimed to tackle the need for mental health care in the general population, but recruitment was not limited to those seeking support. Moreover, the number of participants in the trial was not large, due to the relatively short recruitment period, which was essential in order to minimize the effects of contextual factors and rapid changes during a pandemic. Furthermore, it has to be highlighted that the control condition, despite being a “natural” control, was a highly heterogeneous group to contrast EW intervention due to the pandemic context. Finally, this study assessed only one possible direction for EW; thus, the evidence in this paper is limited to the specific instruction for EW that was presented to the participants. Nevertheless, this study, to the best of our knowledge, is the first to assess the effects and the applicability of an EW intervention in the context of the current COVID-19 pandemic.

To understand the effects of the EW intervention, it is important to note that the circumstances under which it was performed could have altered/affected the mechanisms involved in previously reported beneficial effects of EW. Namely, in the vast majority of prior studies, the EW was conducted either after or in absence of a specific traumatic or stressful event. In contrast, in this study, EW was performed at a moment when the stressor was and still is present, without available and reliable information on when it is expected to end. Thus, it is possible that potential integration of experiences, cognitive adaptation, and reorganization or enhanced emotion regulation required for beneficial effects of EW was disrupted under these circumstances. Furthermore, engaging in EW during a stressful event could lead to focusing one’s attention on pandemic-related content and expectations, thus increasing awareness of the potential threat and/or exaggerating the likelihood of negative outcomes. If this is the case, EW interventions could function as rumination or emotional ventilation, which have been shown to be maladaptive mechanisms for coping with stress and trauma (Littleton et al., 2007). Studies exploring the relationship between rumination style and the effects of EW on the reduction of depressive symptoms showed that some ruminative styles moderate effects of EW on depressive symptoms (Sloan et al., 2008). Therefore, it is possible that elevated stress levels following the EW could be attributed to the timing of the intervention.

The second question is content or the topic of EW. In this study, the instruction for EW was to focus on thoughts and feelings related to pandemic. We opted for focusing the EW prompt on pandemic-related experiences, since previous studies showed that EW interventions with more specific instructions had a higher chance of producing positive results (Reinhold et al., 2018). On the other hand, as some authors discussed, participants’ motivation to engage in EW and their need for intervention are fundamental requirements upon which the effectiveness of EW depends (Rude and Haner, 2018). In the context of pandemic, which is undoubtedly stressful, it is possible that some people were not motivated or did not have a need for additional intervention as they were coping with the pandemic through avoidance of pandemic-related thoughts or by having supportive social relationships in which they were already emotionally expressive, and thus experienced EW as additional exposure to a source of distress. Thus, the increased levels of stress observed in our study could be the result of additional exposure to already widely present pandemic-related content that does not allow enough time for habituation and can interfere with the alternation of intrusion and avoidance, which characterizes natural processing of stressful or traumatic events (Van Emmerik et al., 2002). As our study design does not allow for inference on the effects of the specificity of the EW instruction, future research should explore if the EW intervention would be beneficial if focused on the content not related to the source of distress, or if focused on life after the stressful events are over.

Moreover, our data suggest that EW, when administered online, may not be equally appealing to different age and gender groups. Specifically, the increased stress was recorded among men and among those of age ≥30 years. One could argue that these differences could be attributed to different levels of digital literacy, but we do not believe that is the case here. Namely, it is more likely that digital literacy presents a limiting factor to population-wide implementation of any online interventions and that for those who volunteer to participate in online mental health interventions, the online format does not represent a barrier. It is more likely that age and gender play an important role in how natural and appealing they perceive EW to be and consequently how engaging and immersive they find it. In line with that, previous studies found that participants’ characteristics and individual differences (Sabo Mordechay et al., 2019), as well as mental health status (Pavlacic et al., 2019), moderate the effects of EW. Therefore, the negative effects of EW on some of the outcome variables obtained at the group level could be attributed to rather broad inclusion criteria for participants and, more specifically, to negative effects on certain subgroups of participants.

It should, however, be noted that despite not leading to symptom relief, the majority of participants from the intervention group in our study reported that the experience of participating in the research was personally useful to them. Similarly, as in a study by Lange-Nielsen and colleagues (Lange-Nielsen et al., 2012), although it did not result in a measurable improvement in health, participants who went through the EW intervention found that experience a meaningful process. These insights and beneficial experiences reported by participants should not be overlooked either. As stated by some authors, “feeling better” during the disaster should not be taken for granted and should be considered a desirable outcome irrespective of any longer-term benefits (Wessely and Deahl, 2003).

Therefore, our study does not necessarily suggest abandonment of EW interventions as such, but rather recommends tailoring specific EW intervention modalities in accordance with individual needs, and using EW interventions as a part of a comprehensive stress management approach. EW may help people to better understand a stressful experience, be mentally prepared for the trauma-related difficulties, or prevent rumination (Kleim et al., 2015; Sloan et al., 2008). However, its usage as a stand-alone intervention for the reduction of psychological distress during pandemic was not supported by our study.

## Conclusion and Directions for Future Research

We assessed the effectiveness of a 2 week EW intervention for the reduction of depression, anxiety, and stress symptoms in the general population during the COVID-19 pandemic. Despite EW being recognized as a beneficial intervention in a variety of different settings, our trial showed that, when applied in the context of the COVID-19 pandemic, not only does it not benefit one’s mental health, but it may actually be harmful and lead to increased symptoms of stress. Our trial suggests that EW may be particularly harmful to older and male participants. Our data strongly indicate that one should be highly cautious when applying EW or similar self-guided interventions in novel contexts, especially during highly stressful and unpredictable times. It might be the case that under such circumstances, clinical supervision and guidance are necessary for EW to be effective. Furthermore, considering individual differences and the motivation to participate in this type of intervention may result in a more selective but effective approach to remedy pandemic-related stress. Finally, it is worth assessing how differently directed EW interventions may prove to be more efficient.

## Data Availability Statement

The raw data supporting the conclusions of this article will be made available by the authors, without undue reservation.

## Ethics Statement

The studies involving human participants were reviewed and approved by Institutional Ethics Committee of the Department of Psychology, Faculty of Philosophy, University of Belgrade Protocol number 2020/20. The patients/participants provided their written informed consent to participate in this study.

## Author Contributions

All authors contributed equally to the conception and the design of this work. MV and JB collected and analyzed the data. MV drafted the manuscript. JB and SP critically reviewed and revised the manuscript. All authors read and approved the final version submitted for publishing.

## Conflict of Interest

The authors declare that the research was conducted in the absence of any commercial or financial relationships that could be construed as a potential conflict of interest.
