# Supplementary material for: Effectiveness of Expressive Writing in the Reduction of Psychological Distress During the COVID-19 Pandemic: A Randomized Controlled Trial
Source: Front Psychol. 2020 Nov 10;11:587282. doi: 10.3389/fpsyg.2020.587282 (PMC7683413; doi:10.3389/fpsyg.2020.587282)
Supplement: Supplementary file 1 [file Data_Sheet_1.pdf]

Supplementary Material

Results of per protocol analysis

Table 1. Sample description in per protocol analysis

Variable		Treatment	Control
Gender	Male (n, %)	9 (26.5%)	14 (28.0%)
	Female (n, %)	25 (73.5%)	36 (72.0%)
Age (M, SD)		32.50 (9.526)	32.24 (10.627)
Years of education	(M, SD)	15.42 (3.336)	15.57 (2.424)

Table 2. Outcome measures and effects of intervention in per protocol analysis

	Treatment (N=37)				Control (N=53)			
Outcomes	Baseline M (SD)	Post-treatment M (SD)	Difference	r	Baseline M (SD)	Post-treatment M (SD)	Difference	r
DASStotal	19.08 (13.183)	20.89 (12.080)	t(36)=-0.854, p=.399	.481	15.55 (11.954)	13.30 (10.608)	t(52)=1.873, p=.067	.707
Depression (DASS)	4.92 (4.548)	5.64 (4.951)	t(36)=-0.884, p=.382	.444	4.40 (4.797)	3.68 (3.902)	t(52)=1.679, p=.099	.763
Anxiety (DASS)	4.57 (4.463)	4.27 (4.544)	t(36)=0.354, p=.725	.357	3.00 (3.937)	2.66 (3.578)	t(52)=0.850, p=.399	.704
Stress (DASS)	9.59 (6.053)	10.97 (5.003)	t(36)=-1.879, p=.068	.689	8.15 (5.040)	6.96 (5.211)	t(52)=1.993, p=.051	.642
Wellbeing (WHO)	3.15 (0.828)	3.22 (0.800)	t(36)=-0.824 p=.416	.797	3.16 (0.828)	3.29 (0.819)	t(52)=-1.504, p=.139	.732
SQOL	4.88 (1.017)	5.02 (1.034)	t(36)=-1.337, p=.190	.802	4.96 (1.155)	5.00 (1.120)	t(52)=-0.352, p=.727	.745
Hard time making it through the pandemic	2.00 (0.943)	2.05 (1.026)	t(36)=-0.291, p=.773	.345	1.70 (0.749)	1.53 (0.696)	t(52)=1.766, p=.083	.533

The ANCOVA for treatment versus control group on post-test DASS total scores, controlling for baseline DASS score was found to have statistically significant main effect of group $F(1,87) = 8.450$, $p = .005$, ${}_{p}\eta^2 = .089$. There is a significant effect of treatment on posttest Depression after controlling for depression at baseline $F(1,87) = 5.046$, $p = .027$, ${}_{p}\eta^2=.055$ and Stress, after controlling for stress at baseline $F(1,87) = 15.046$, $p < .001$, ${}_{p}\eta^2=.137$, with treatment group scoring higher on all measures. Main effect of group on posttest Anxiety, after controlling for baseline score was not found $F(1,87) = 1.188$, $p = .279$, ${}_{p}\eta^2 = .013$. Main effect of group on Wellbeing and Subjective quality of life post-test scores, when controlling for baseline scores on these scales was not found either $F(1,87) = 0.263$, $p = .609$, ${}_{p}\eta^2 = .003$, $F(1,87) = 0.303$, $p = .584$, ${}_{p}\eta^2 = .003$ respectively. The ANCOVA for treatment *vs* control group on post-test measure of having a hard time making it through the coronavirus pandemic and state of emergency, when controlling for baseline score on this measure showed significant effect of group, with treatment group scoring significantly higher than control group $F(1,87) = 5.551$, $p = .021$, ${}_{p}\eta^2 = .060$.
